# Emphysematous cystitis presenting as severe confusion and abdominal pain: two case reports

**DOI:** 10.1186/s13256-015-0530-y

**Published:** 2015-03-07

**Authors:** Vincent De Coninck, Dirk Michielsen

**Affiliations:** Department of Urology, University Hospital (UZ), Laarbeeklaan 101, 1090 Brussels, Belgium

**Keywords:** Computed tomography, Diabetes mellitus, Emphysematous cystitis, Pneumaturia, Urinary tract infection

## Abstract

**Introduction:**

Emphysematous cystitis is a very rare complicated urinary tract infection characterized by air in the bladder wall.

**Case presentation:**

We report two clinical cases of emphysematous cystitis of an 83-year-old Caucasian woman with diabetes mellitus and a 78-year-old Caucasian man with no past medical history. They presented with severe confusion and abdominal distension. Emphysematous cystitis was diagnosed in time with a thorough physical examination, urine analysis and computed tomography. The patients were successfully treated with antibiotic therapy and bladder drainage.

**Conclusion:**

This rare disorder should be recognized in time and treated properly to guarantee survival.

## Introduction

Emphysematous cystitis is a very rare urinary tract infection. Two cases presenting as severe confusion and abdominal pain are described. Patients should be diagnosed in time by performing a thorough physical examination and selecting accurate diagnostic tests.

## Cases presentation

### Case one

An 83-year-old Caucasian woman was referred by her general practitioner to our emergency department due to agitation and hallucination. Because of pyuria and haematuria on dipsticks, the general practitioner started ciprofloxacin 5 days earlier.

She had a medical history of Parkinson disease, atrial flutter, rheumatoid arthritis and diabetes mellitus.

A physical examination revealed severe confusion, dehydration, abdominal distension and suprapubic tenderness.

Blood analysis revealed prerenal acute kidney failure (creatinine 2.24mg/dL, urea 98mg/dL), signs of infection (C-reactive protein 30mg/dL, serum white blood count 17200/mm^3^) and a well-controlled glycaemia (glycated haemoglobin, A1c, 48mmol/mol).

Urine analysis disclosed leukocyturia, bacteriuria and 540 red blood cells per field.

**Figure 1:**
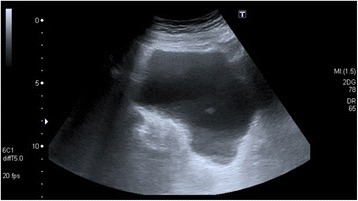
**Ultrasound of the bladder demonstrating irregular thickening of the bladder wall with extensive intramural echogenic foci.**

Because of the abdominal findings, an abdominal ultrasonography was performed. This showed irregular thickening of her bladder wall with extensive intramural echogenic foci (Figure 1).

**Figure 2:**
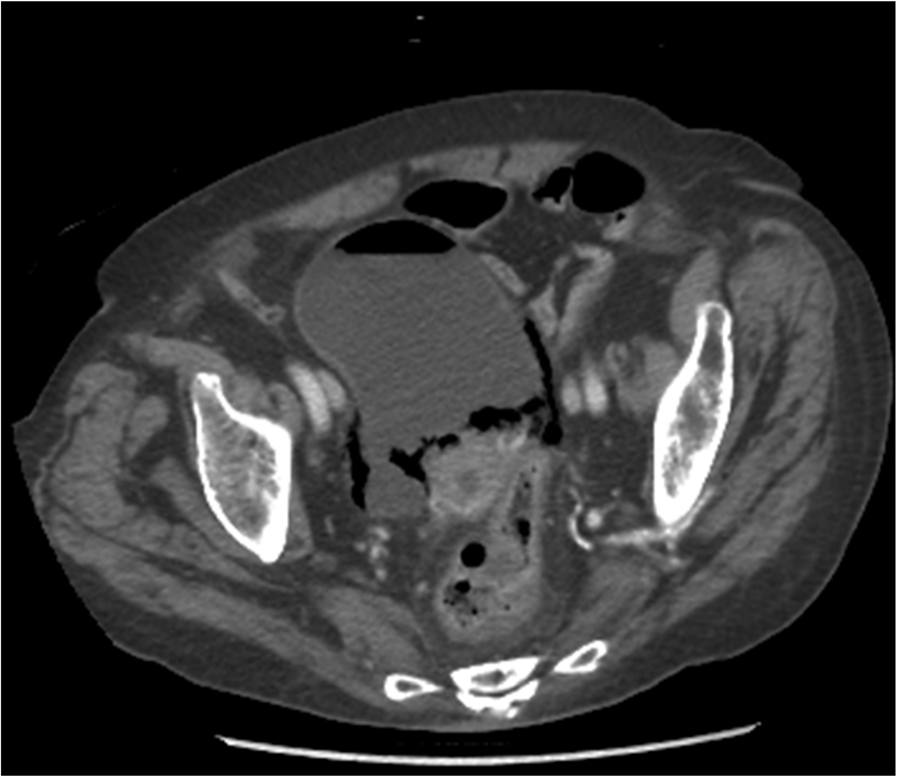
**Computed tomography.** Axial view showing intraluminal air and emphysematous changes of the bladder wall.

In addition, a computed tomography was performed. This showed intraluminal air and emphysematous changes of her bladder wall (Figure 2).

Parenteral ciprofloxacin was continued and a 16 Fr Foley catheter was inserted for optimal drainage. After catheterization, pneumaturia was observed.

**Figure 3:**
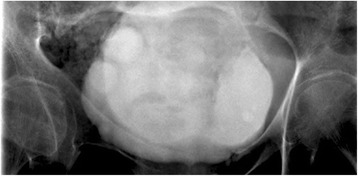
**Retrograde cystography revealing air at the posterior surface of the bladder and an irregular bladder wall with delineated radiolucent areas.**

A retrograde cystography was performed to exclude fistulas. This showed air at the posterior surface of her bladder and an irregular bladder wall with delineated radiolucent areas (Figure 3).

**Figure 4:**
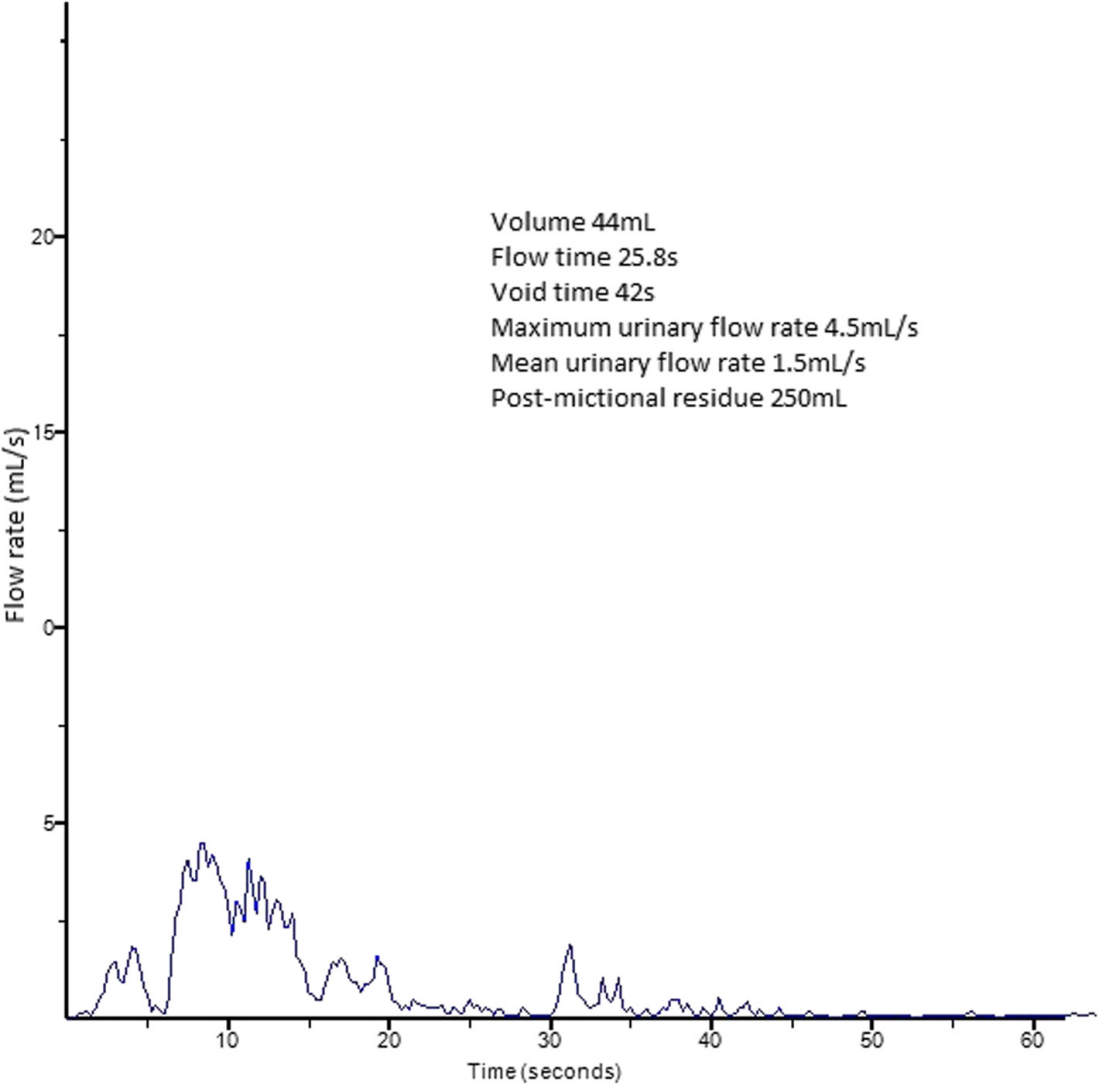
**Uroflowmetry revealing a micturition of 44mL with a maximum flow of 4.5mL/second.**

To exclude outlet obstruction, a uroflowmetry was done. This examination revealed a micturition of 44mL with a maximum flow of 4.5mL/second. The contribution of this examination was limited because of the low micturition volume. Post-mictional residue was 250mL (Figure 4).

**Figure 5:**
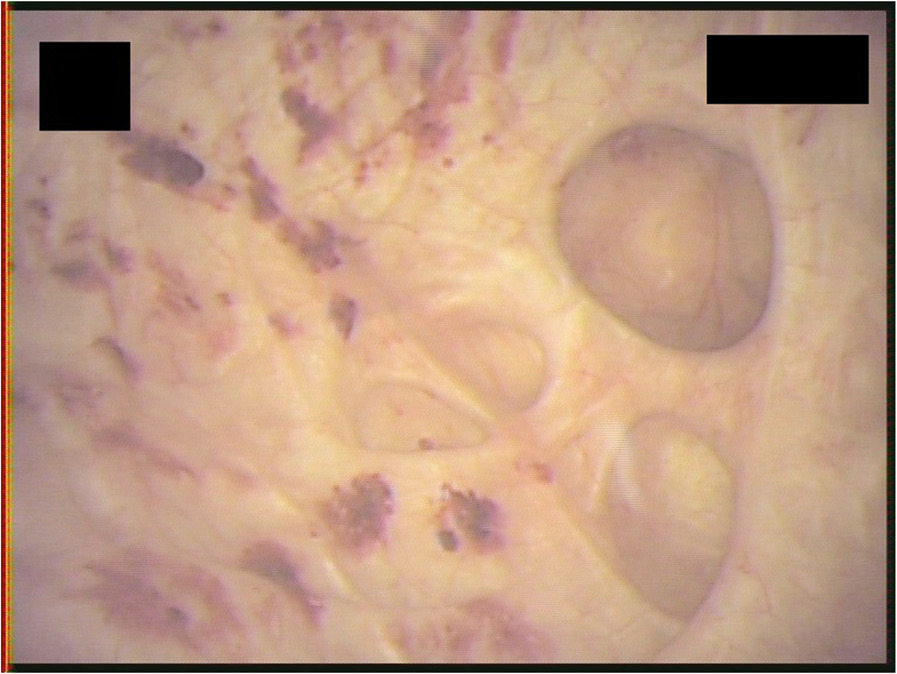
**Cystoscopy revealing inflammatory lesions, diverticula and trabeculation.**

Cystoscopy revealed inflammatory lesions, diverticula and trabeculation (Figure 5).

After 3 days, *Escherichia coli* resistant to ciprofloxacin but sensitive to temocillin was cultured at more than 1×10^6^ colony-forming units/mL, so intravenous antibiotics were switched to the latter.

**Figure 6:**
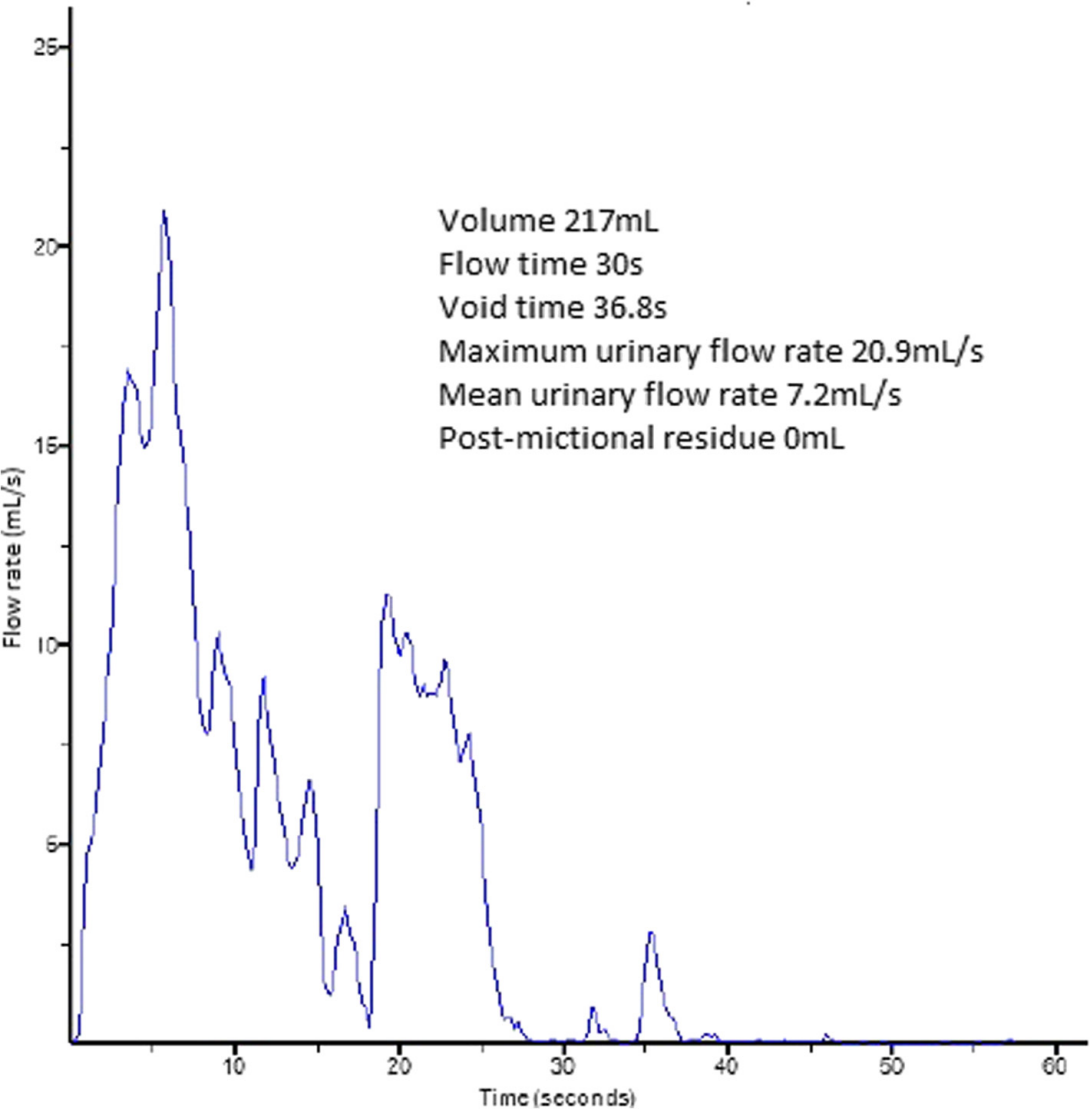
**Revealing a micturition of 217mL with a maximum flow of 20.9mL/second.**

After 10 days she recovered. Her bladder catheter was removed and antibiotics were stopped. A repeat urine culture was sterile and she was sent home with clean intermittent catheterization. Nitrofurantoin was prescribed in the prophylaxis of urinary tract infections. A follow-up consultation at 4 weeks was arranged for uroflowmetry control. This examination revealed bladder function recovery with no post-mictional residue, so clean intermittent catheterization was stopped (Figure 6).

### Case two

**Figure 7:**
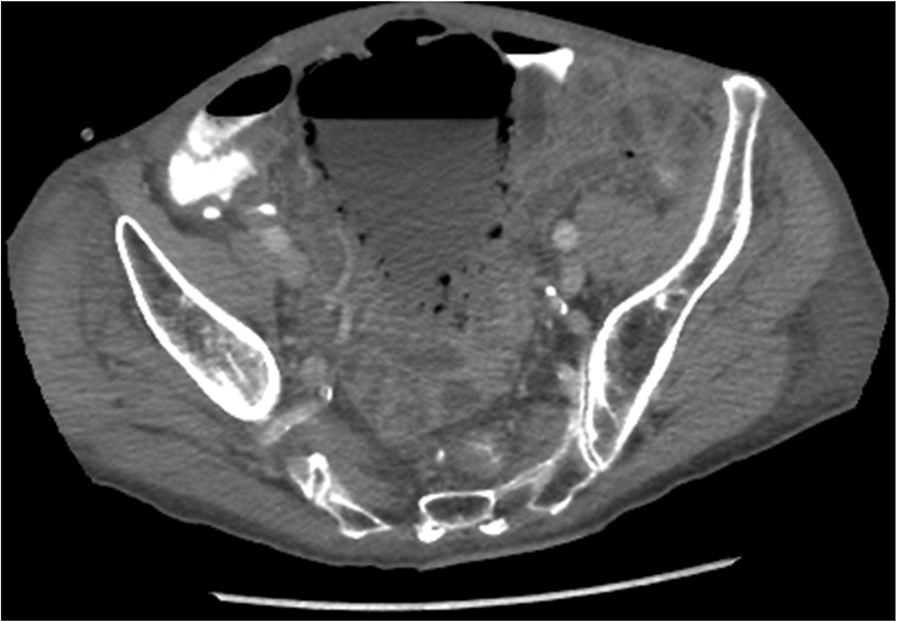
**Computed tomography.** Axial view showing multiple gas foci in a diffuse collection of gas within the thickened bladder wall.

**Figure 8:**
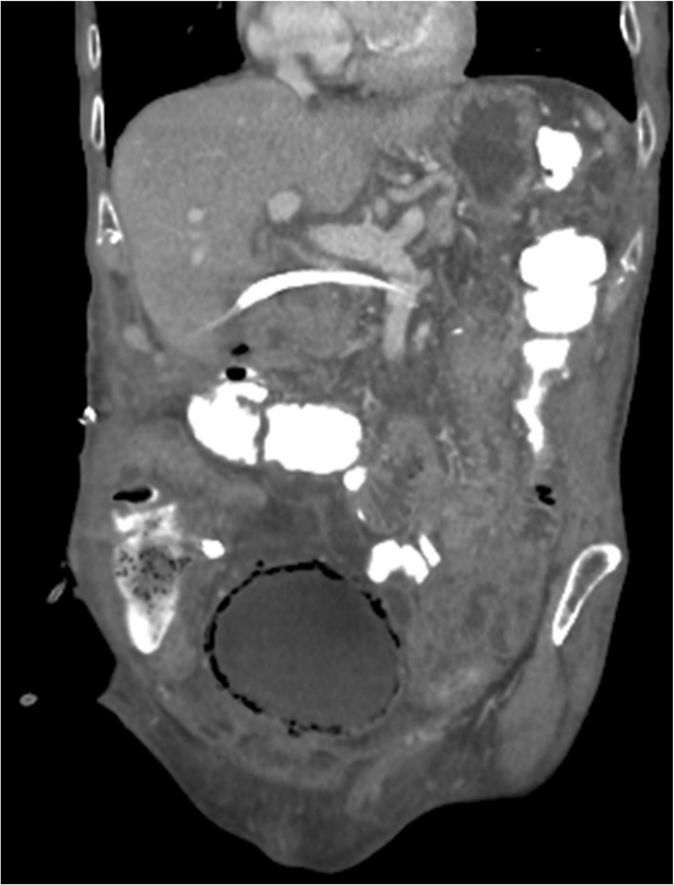
**Computed tomography.** Coronal view revealing emphysematous changes in the bladder wall.

A 78-year-old Caucasian man with an adenocarcinoma of the pancreas was hospitalized for a pancreaticoduodenectomy. He has no past medical history. Four days after surgery, the bladder catheter was removed with subsequent normal micturition. One week after the Whipple procedure, he became aggressive, had severe confusion and abdominal pain. Abdominal palpation revealed diffuse tenderness without peritoneal signs. Laboratory testing showed a serum white blood count of 20200/mm^3^. Urine analysis showed significant leukocyturia and bacteriuria without haematuria. Because of the disturbing abdominal examination, abdominopelvic computed tomography was performed. This revealed emphysematous changes in his bladder wall (Figures 7 and 8).

Intravenous ciprofloxacin was started and a bladder catheter was replaced. After catheterization, pneumaturia was not observed. After 3 days, more than 1×10^6^ colony-forming units/mL *E. coli* sensitive to ciprofloxacin was cultured. His abdominal pain and confusion disappeared after 2 days. After 1 week of treatment with antibiotics and bladder catheterization, his bladder wall returned to normal on computed tomography scanning of his abdomen.

## Conclusions

Emphysematous cystitis is a rare disorder characterized by air in the bladder wall and lumen. The triggers and cellular mechanisms of this complicated urinary tract infection are unclear. Gas in the bladder wall is probably produced by natural fermentation of glucose by bacteria.

Older women with uncontrolled diabetes are at highest risk of developing emphysematous cystitis. A 2:1 female to male predominance is noticed and two-thirds of reported cases are diabetics. However, this complicated urinary tract infection should not be overlooked in non-diabetic male patients as presented in the second case. Other risk factors comprise urinary tract obstruction, indwelling urinary catheters, chronic urinary tract infections, neurogenic bladder or immunosuppression [1].

Presentation can vary from asymptomatic to severe sepsis. More than half of patients present with abdominal pain, classic symptoms of acute cystitis and pneumaturia. Although pneumaturia appears to be a highly specific symptom, it is a rare patient complaint [2]. However, pneumaturia was observed after bladder catheterization in the first case. Both patients presented with severe confusion. This atypical symptom should always be further investigated as it can be the only sign of severe illness.

Urine analyses and Gram staining are essential in managing patients with cystitis emphysematosa. Laboratory testing usually reveals positive urine cultures with *E. coli* or *Klebsiella pneumoniae* involving 80% of cases [3]. In the first case accurate treatment was delayed because of *E. coli* resistance to ciprofloxacin.

The severity and extent of emphysematous cystitis is best defined by computed tomography. Because of the increased use of computed tomography, emphysematous cystitis is often incidentally diagnosed. Images are characterized by multiple gas foci in a diffuse collection of gas within the thickened bladder wall.

When performing ultrasonography or plain films of the abdomen, air surrounding the bladder can be seen in most cases.

Cystoscopy may show submucosal emphysema and can be suggestive for bladder outlet obstruction.

The impeded flow from the bladder to the urethra can be confirmed by uroflowmetry.

Cystography can show irregular thickening of the bladder wall, representing submucosal blebs of air. Furthermore, it is the most sensitive imaging modality to exclude other causes of pneumaturia like enterovesical fistula [4].

In most cases, this gas-forming infection can be treated with antibiotics and bladder catheterization for optimal drainage. Of course, underlying medical conditions and glycaemic levels in diabetics should be controlled as well. In case of haematuria that includes blood clots or necrotizing tissue, bladder irrigation or transurethral debridement might be necessary. Emphysematous cystitis seldom requires (partial) cystectomy [2].

If not diagnosed in time, cystitis emphysematosa may result in urosepticaemia and even in death in up to 7% of patients [5].

## Consent

Written informed consent was obtained from the patients for publication of this case report and accompanying images. A copy of the written consents is available for review by the Editor-in-Chief of this journal.
